# Ocular biometric characteristics of cataract patients in western China

**DOI:** 10.1186/s12886-018-0770-x

**Published:** 2018-04-17

**Authors:** Qing Huang, Yongzhi Huang, Qu Luo, Wei Fan

**Affiliations:** Department of Ophthalmology, West China Hospital of Sichuan University, Sichuan Province, Chengdu, China

**Keywords:** Axial length, Anterior chamber depth, Keratometric power, Corneal astigmatism, Myopia, IOL master

## Abstract

**Background:**

We aimed to measure ocular biometric characteristics in older cataract patients from western China.

**Methods:**

Ocular biometry records were retrospectively analyzed for 6933 patients with cataracts (6933 eyes) at least 50 years old who were treated at West China Hospital of Sichuan University.

**Results:**

Partial coherence laser interferometry gave the following population averages: axial length (AL), 24.32 ± 2.42 mm; anterior chamber depth (ACD), 3.08 ± 0.47 mm; keratometric power (K), 44.23 ± 1.66 diopters; and corneal astigmatism (CA), 1.00 ± 0.92 diopters. The percentage of individuals with AL > 26.5 mm was 13.66%, while the percentage with CA > 1.0 diopters was 35.54%. Mean AL and ACD showed a trend of decrease with increasing age (*P* < 0.001). AL correlated positively with ACD (Spearman coefficient, 0.542) and CA (0.111), but negatively with K (− 0.411) (all *P* < 0.01). K also correlated negatively with ACD (− 0.078, *P* < 0.01).

**Conclusions:**

These results show, for the first time, that older cataract patients from western China have similar ocular biometric characteristics as other populations. The high prevalence of severe axial myopia warrants further investigation.

## Background

Cataract surgery is the most commonly performed surgical procedure worldwide. Advances in surgical instruments and intraocular lens design have increased the expectations of surgeons and patients for satisfactory postoperative refractive results. To achieve this goal, accurate biometric measurements are crucial. The ocular biometric characteristics of a population, including axial length (AL), anterior chamber depth (ACD), keratometric power (K) and corneal astigmatism (CA), are important to know in order to help engineers design intraocular lenses and to help surgeons select the most appropriate lenses for patients. Partial coherence laser interferometry is used most frequently in the clinic to determine these parameters, and it is routinely used to calculate lens implant power for cataract surgery [1, 2].

Studies have described ocular biometric characteristics of various populations [3–12], including several from China [10–12]. To our knowledge, the values of ocular biometry parameters have never been published for populations from western China. The present work provides the first hospital-based population study of ocular biometric characteristics of cataract patients 50 yr. and older from western China.

## Methods

Medical records were retrospectively reviewed for a consecutive series of patients aged 50 years and older with cataracts treated at West China Hospital of Sichuan University (Chengdu, China) from November, 2011 to August, 2014. Patients who lived outside western China or who underwent photorefractive surgery were excluded, as were patients with retinal detachment, eye trauma, eyeball atrophy, or severe cataracts that could not be analyzed using the IOL Master device. The study was carried out in accordance with the Declaration of Helsinki. The study protocol was approved by the Ethics Committee of the West China Hospital of Sichuan University (2016–324), and procedures were performed in accordance with relevant guidelines and regulations.

Ocular biometry was performed using the IOL Master system (IOL Master 500, Carl Zeiss Meditec AG, Jena, Germany), which uses signals from the tear film and retinal pigment epithelium to measure AL. AL measurements were performed a minimum of five times in each eye, the AL was obtained from the composite mean value of five measurements. ACD was defined as the distance from the anterior corneal surface to the anterior lens surface, and it was measured a minimum of five times. Minimal K (K1) and maximal K (K2) were determined at the maximal and minimal radii of curvature, and the two values were averaged to obtain K. Keratometric measurements were also taken three times. CA was calculated as the absolute difference between K values at the two meridians. We classified astigmatism as “with-the-rule” (WTR) when the steep meridian on the corneal surface was 60–120 degrees, or “against-the-rule” (ATR) when the steep meridian was 0–30 degrees or 150–180 degrees [10, 13]. Other cases of astigmatism were classified as oblique. Since biometric characteristics were similar within pairs of eyes [3], the right or left eye of each patient was randomly selected for all analyses. Similar results were obtained when data from only the right eyes of all patients were used (data not shown).

Statistical analyses were performed using SPSS 19.0 (IBM, USA). Only patients with complete data were included in analyses. Normality of data was tested for all variables using the Kolmogorov-Smirnov (K-S) test, which was taken to indicate skewed distribution if *P* < 0.05. Differences between two groups were assessed for significance using the *t* test if data were normally distributed, and the Mann-Whitney U test otherwise. Differences among more than two groups were assessed using analysis of variance (ANOVA) followed by the least-squares difference post-hoc test when data were normally distributed. When data were skewed, differences were assessed using the Kruskal-Wallis test. Severe axial myopia was defined as AL longer than 26.5 mm in this study. Differences between groups in the percentage of severe axial myopia were assessed using the Pearson chi-squared test. Possible correlations between biometric parameters were assessed using Spearman’s correlation coefficient, while possible associations among AL, ACD, K and CA were assessed using polynomial regression. The threshold of significance was defined as *P* < 0.05.

## Results

The final study population included 6933 cataract patients from western China (3311 men, 3622 women) ranging in age from 50 to 98 years. The population was divided into four age groups: 50–59 yr., 17.73% of the total population, mean age 55.40 ± 2.98 yr.; 60–69 yr., 35.41%, 64.68 ± 2.92 yr.; 70–79 yr., 34.50%, 74.12 ± 2.78 yr.; and 80+ yr., 12.36%, 83.22 ± 3.84 yr.

### Population distribution of ocular biometry characteristics

**Fig. 1:**
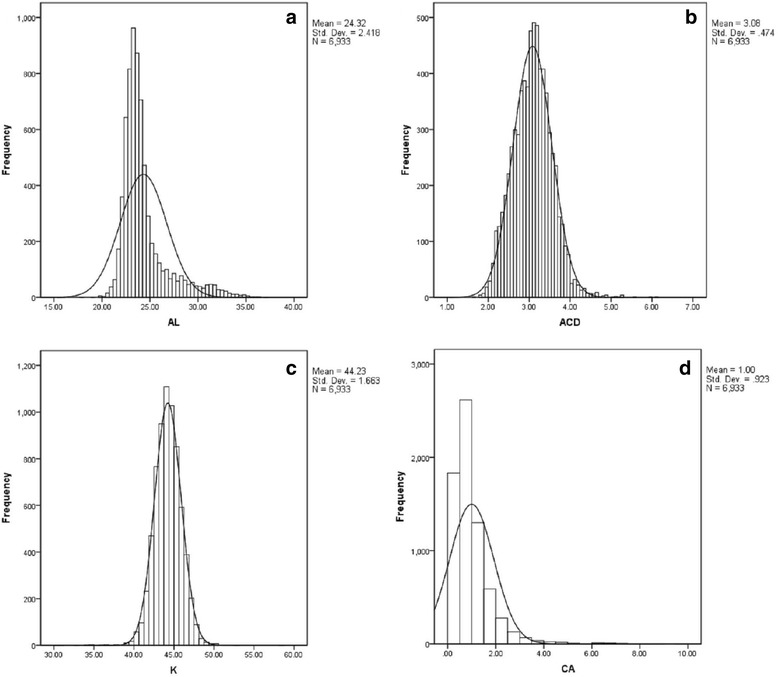
Distribution of ocular biometric characteristics in a western Chinese cataract patient population. (**a**) Distribution of axial length (AL) was positively skewed and peaked. (**b**) Distribution of anterior chamber depth (ACD) was normal. (**c**) Distribution of keratometric power (K) was negatively skewed. (**d**) Distribution of corneal astigmatism (CA) was positively skewed and peaked

Figure 1 shows the distribution of ocular biometric characteristics across the entire study population. The AL distribution (mean, 24.32 ± 2.42 mm) was positively skewed (1.914) and peaked, with kurtosis of 3.963 (K-S test, *P* < 0.001). The ACD distribution (mean, 3.08 ± 0.47 mm) was normal (K-S test, *P* = 0.097). The K distribution (mean, 44.23 ± 1.66 diopters [D]) was negatively skewed (− 0.29), with kurtosis of 3.611 (K-S test, *P* < 0.001). The CA distribution (mean, 1.00 ± 0.92 D) was skewed towards the right (5.485) and strongly peaked, with kurtosis of 73.32 (K-S test, *P* < 0.001).

### Distribution of ocular biometry characteristics by sex

Table 1 shows ocular biometry characteristics stratified by sex. Mean AL was significantly longer in men (24.79 ± 2.48 mm) than in women (23.88 ± 2.27 mm, *P* < 0.001), and ACD was deeper in men (3.16 ± 0.47 vs 3.01 ± 0.47 mm, *P* < 0.001). Conversely, mean K was significantly greater in women (44.56 ± 1.61 vs 43.87 ± 1.65 D, *P* < 0.001). Mean CA was similar between men and women (0.99 ± 0.84 vs 1.0 ± 0.99 D, *P* = 0.398).

**Table 1:** Ocular biometric characteristics of western Chinese adult cataract patients, stratified by sex

Gender	AL (mm)	ACD (mm)	K (D)	CA (D)
Male (*n* = 3311)	24.79 ± 2.48	3.16 ± 0.47	43.87 ± 1.65	0.99 ± 0.84
Female (*n* = 3622)	23.88 ± 2.27	3.01 ± 0.47	44.56 ± 1.61	1.0 ± 0.99
Total (*n* = 6933)	24.32 ± 2.42	3.08 ± 0.47	44.23 ± 1.66	1.0 ± 0.92
P^a^	< 0.001^b^	< 0.001^c^	< 0.001^b^	0.398^b^

^a^Difference between males and females

^b^Mann-Whitney U-test

^c^*t* test

### Distribution of ocular biometry characteristics by age

**Fig. 2:**
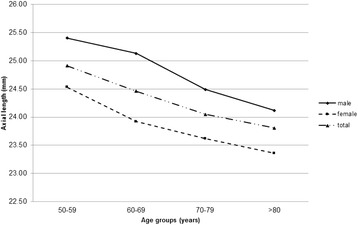
Distribution of axial length across the four age groups of the cataract population. Mean axial length showed a decreasing trend with age

**Fig. 3:**
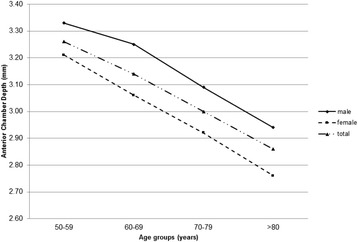
Distribution of anterior chamber depth across the four age groups of the cataract population. Mean anterior chamber depth showed a decreasing trend with age

**Fig. 4:**
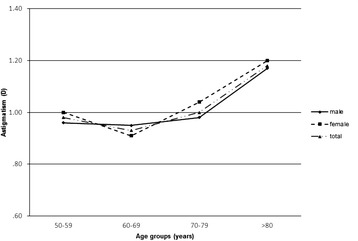
Distribution of corneal astigmatism across the four age groups of the cataract population. Mean corneal astigmatism did not vary with age

Mean AL and ACD showed a trend of decrease with increasing age (both P < 0.001; Table 2, Figs. 2 and 3). While CA varied significantly between some age groups (*P* < 0.001; Table 2, Fig. 4), there were no differences between others (age group 50–59 and 60–69, *P* = 0.143; group 50–59 and 70–79, *P* = 0.091), suggesting there was no trend change with age. The percentage of patients with AL > 26.5 mm also showed a trend of decrease with increasing age (*P* < 0.001, Table 3). The percentage was 13.66% across the entire population (Table 3), and it rose to 21.72% in the youngest patient group (50–59 years). Furthermore, 9.29% of all patients had AL > 28 mm (Table 4). In addition, it was noted that, in the group of patients with AL > 26.5 mm, male accounted for 58.82% (Table 3).

**Table 2:** Distribution of ocular biometric parameters by age group and sex

Age & sex	N	AL (mm)	ACD (mm)	K (D)	CA (D)
50–59 yr					
Male	535	25.4 ± 3.07	3.33 ± 0.44	43.66 ± 1.76	0.96 ± 0.88
Female	694	24.53 ± 2.98	3.21 ± 0.44	44.37 ± 1.83	1.0 ± 1.18
Total	1229	24.91 ± 3.05	3.26 ± 0.45	44.06 ± 1.83	0.98 ± 1.06
60–69 yr					
Male	1095	25.13 ± 2.68	3.25 ± 0.44	43.96 ± 1.55	0.95 ± 0.85
Female	1360	23.92 ± 2.29	3.06 ± 0.45	44.66 ± 1.53	0.91 ± 0.89
Total	2455	24.46 ± 2.54	3.14 ± 0.46	44.31 ± 1.58	0.93 ± 0.88
70–79 yr					
Male	1183	24.49 ± 2.12	3.09 ± 0.46	44.01 ± 1.5	0.98 ± 0.79
Female	1209	23.62 ± 1.87	2.92 ± 0.45	44.65 ± 1.69	1.04 ± 0.99
Total	2392	24.05 ± 2.04	3.0 ± 0.46	44.28 ± 1.61	1.0 ± 0.9
> 80 yr					
Male	498	24.12 ± 1.76	2.94 ± 0.45	43.87 ± 1.65	1.17 ± 0.88
Female	359	23.36 ± 1.44	2.76 ± 0.43	44.56 ± 1.61	1.2 ± 0.9
Total	857	23.81 ± 1.67	2.86 ± 0.45	44.23 ± 1.61	1.18 ± 0.89
P^a^		< 0.001	< 0.001	0.004	< 0.001

^a^Difference between age groups

**Table 3:** Percentages of cataract patients with axial length > 26.5 mm in each age group

Age (yr)	n1^a^	n2^b^	n1/n2 (%)	Nm^d^	Nf^e^	Nm/n1 (%)
50-59	267	1229	21.72	140	127	52.43
60–69	381	2455	15.52	230	151	60.37
70–79	249	2392	10.41	153	96	61.45
> 80	50	857	5.83	34	16	68.0
Total	947	6933	13.66	557	390	58.82
P^c^			< 0.001			

^a^no. with axial length > 26.5 mm in this age group

^b^Total N in this age group

^c^Difference between age groups

^d^No. of male with axial length > 26.5 mm in this age group

^e^No. of female with axial length > 26.5 mm in this age group

**Table 4:** Distribution of anterior chamber depth (ACD) across patient subgroups with different axial lengths (AL)

AL (mm)	*N* (%)	ACD (mm)
< 22	348 (5.02)	2.6 ± 0.41
22–25	5069 (73.11)	3.01 ± 0.43
25–28	872 (12.58)	3.38 ± 0.4
> 28	644 (9.29)	3.51 ± 0.41
Total	6933	3.08 ± 0.47
P^a^		< 0.001

^a^Difference between groups

### Corneal astigmatism: Severity, type and variation with age

**Fig. 5:**
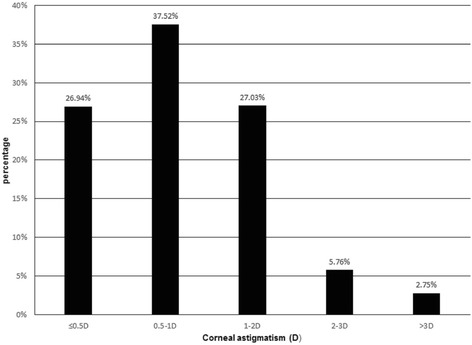
Distribution of corneal astigmatism (in diopters, D) across the entire cataract population

**Fig. 6:**
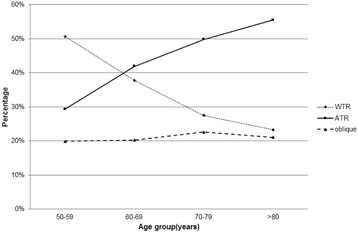
Distribution of corneal astigmatism axes across the four age groups of the cataract population. Across all age groups, the prevalence of oblique astigmatism was lower than that of with-the-rule (WTR) or against-the-rule (ATR) astigmatism. The prevalence of WTR astigmatism showed a decreasing trend with age, whereas the prevalence of ATR astigmatism showed an increasing trend with age

Corneal astigmatism was less than 0.5 D in 26.94% of eyes, 0.5–1.0 D in 37.52%, 1.0–2.0 D in 27.03%, 2.0–3.0 D in 5.76%, and > 3.0 D in 2.75% (Fig. 5). It was WTR in 34.76% of eyes, ATR in 44.09%, and oblique in 21.15%. The distributions of astigmatism axes varied significantly with age (*P* < 0.001, Fig. 6). The percentage of patients with WTR astigmatism decreased with age, while the percentage with ATR astigmatism increased with age.

### Correlations between ocular biometric characteristics

**Fig. 7:**
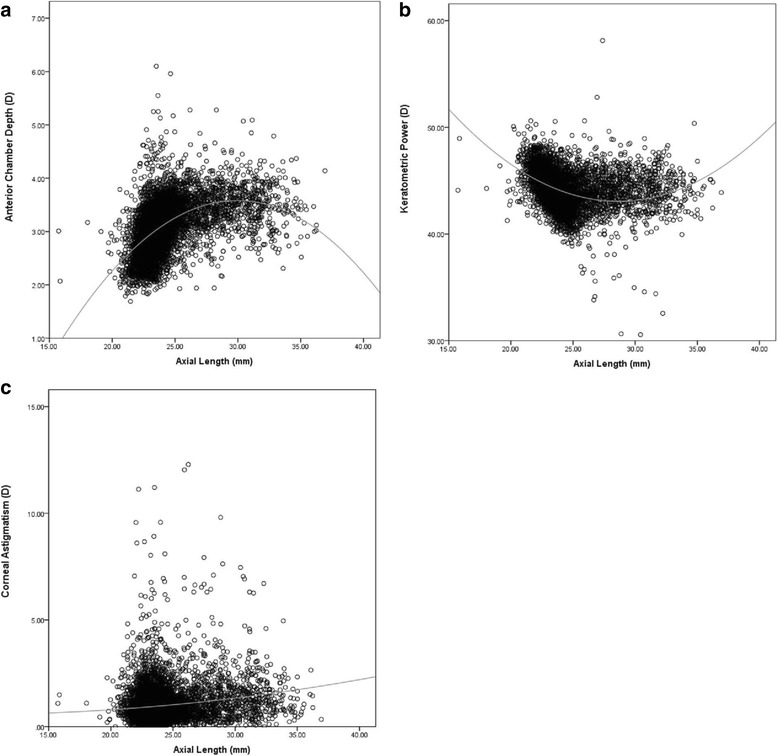
Correlation of axial length with anterior chamber depth, corneal power or corneal astigmatism. (**a**) Anterior chamber depth increased with axial length up to a length of 29.9 mm. (**b**) Keratometric power decreased with increasing axial length up to a length of 28.6 mm. (**c**) Corneal astigmatism increased with axial length over the observed range of lengths

AL correlated positively with ACD (Spearman coefficient, 0.542) and with CA (0.111), but negatively with K (− 0.411) (all *P* < 0.01; Table 5). K correlated negatively with ACD (− 0.078, *P* < 0.01) and positively with CA (0.054, *P* < 0.01). ACD correlated negatively with CA (− 0.003, *P* = 0.79). ACD showed an increasing trend when AL fell below 29.9 mm (Fig. 7a), while K showed a decreasing trend when AL fell below 28.6 mm (Fig. 7b). CA increased with AL (Fig. 7c).

**Table 5:** Pairwise correlations among axial length (AL), anterior chamber depth (ACD), keratometric power (K) and corneal astigmatism (CA)

	ACD (mm)	K (D)	CA (D)
AL (mm)	0.542^a^	− 0.411^a^	0.111^a^
ACD (mm)	–	− 0.078^a^	− 0.003^b^
K (D)	–	–	0.054^a^

^a^a < 0.01

^b^*P* = 0.79

## Discussion

Few studies have focused on large populations of Chinese cataract patients, especially from western China. This study aimed to describe a hospital-based distribution of ocular biometric parameters in western Chinese cataract patients. As most patients came from Sichuan which is the most heavily populated province in western China, the results may be representative, to some extent, of the larger population of western China. In addition to providing a rich description of ocular characteristics to serve as a reference for future work, we found a high prevalence of severe axial myopia, particularly in relatively younger patients, which merits further study.

The AL distribution in our population showed positive skew and significant kurtosis, similar to that observed in a healthy Caucasian population [4], but different from that observed in healthy Singaporean Malays [9]. Mean AL was shorter in our population (24.32 ± 2.42 mm) than in cataract patients from eastern China (24.86 ± 2.72 mm) [14]. The possible explanation may be that the previous study in eastern China [14] included patients aged 18–95 years old; their participants aged 18–49 yr. had longer AL than older participants, making their mean AL longer than ours. At the same time, mean AL was longer in our patients than in cataract patients from southern China (24.07 ± 2.14 mm) [10] and in healthy individuals from southern China (23.48 mm) [15], Europe (23.43 ± 1.51 mm) [5], Latin America (23.38 mm) [6], and other parts of Asia (23.13 ± 1.15 mm [7], 23.13 ± 1.00 mm [8] and 23.55 mm [9]). One likely explanation for these differences between our study and others is ethnicity of Sichuan, which is one of the provinces with most populous ethnic groups of Han Chinese. Another explanation is the relatively high proportion of our cataract patients with severe axial myopia, often defined as AL > 26.5 mm [16, 17]. Our hospital attracts many such patients from Sichuan and other neighbor provinces in western China. Indeed, the proportion of severely axial myopic eyes in our study (13.66%), especially among those aged 50–59 years (21.72%), was higher than in a study of cataract patients from southern China (11.9%) [10]. These results may suggest that patients with severe axial myopia tend to develop cataracts at a younger age and therefore require earlier cataract surgery [10, 18]. In any event, our results are consistent with numerous reports of high global prevalence of myopia. In East Asian countries, this prevalence may reach 70–80% [11, 12, 19, 20], with prevalence of severe myopia ranging from 8.4% to 38% [11, 21, 22]. In Western countries, prevalence of myopia ranges from 25% to 40% [23, 24].

AL in our population showed a gender bias: mean AL was longer in men (24.79 ± 2.48 mm) than in women (23.88 ± 2.27 mm). This echoes findings from studies of cataract populations in the US [25], among Singaporean Chinese [26], and among Chinese from the southern part of the mainland [10, 13]. Our result also echoes findings from studies of healthy eyes in Chinese from the southern part of the mainland [15], Latin Americans [6] and Icelanders [27]. The AL difference between men and women in our study (0.91 mm) was longer than that in the previous studies. This may be caused by the relatively high proportion of male patients with severe axial myopia (58.82% in male vs 41.18% in female). In addition, many cross-sectional studies showed body height was positively correlated with AL, men had taller stature than women, and the association between male gender and longer AL may reflect the difference in stature between the sexes [3, 28–30].

We found that AL showed a trend of decrease with increasing age, as reported in cross-sectional studies from the UK [31, 32], US [33], and southern China [10]. Studies of Singaporeans suggest that this association may reflect that younger generations are generally taller, so it may be a cohort effect associated with improved nutrition [3, 34]. Other studies have proposed that the reduction of AL in the adult eye may serve as an emmetropizing mechanism, correlating with the increase in refractory power [3, 31]. Another possible explanation, at least in our study, is that younger cataract patients are more likely to have axial myopia. Because of the cross-sectional nature of our study, our comparison of different age groups is confounded with generational effects, so prospective studies are needed to determine why AL decreases with age.

ACD showed normal distribution in our population, with greater depth occurring in male patients and younger patients. This is consistent with findings from other studies [8, 9, 13, 15, 26]. This variation of ACD with sex has been attributed to differences in stature, particularly height, between men and women [4, 26]. The variation of ACD with age has been attributed to age-related lens thickening [10, 33], which may shift the iris forward, making the anterior chamber shallower [6]. Our finding of shallower ACD in older women is consistent with the higher prevalence of angle-closure glaucoma in this population [35]. The reported ability of cataract surgery to significantly deepen the anterior chamber [36] implies that older women with cataracts may benefit from earlier surgery [13]. Unfortunately, we could not verify whether cataract surgery deepened the anterior chamber in our population because the IOL Master system does not measure lens thickness or other relative ocular characteristics. Further study is needed to address this question directly.

The K distribution in our population was skewed. K did not clearly increase with age, in contrast to other studies [37, 38], and K was greater in women than men, similar to other studies [5, 9]. Greater mean K in women indicates higher corneal refractory power, which may be an emmetropizing mechanism to compensate for shorter AL.

CA did not increase or decrease with age as other studies [13, 37, 39, 40]. Consistent with the idea that many cataract patients suffer from corneal astigmatism, 35.54% of eyes in our population had CA > 1.0 D, with similarly high proportions reported in Spain (34.8%) [40] and Japan (36.4%) [41]. These results highlight the importance of astigmatism correction during cataract surgery. Toric intraocular lenses can correct corneal astigmatism of 0.7–8.4 D during cataract surgery [40], and such lenses are now widely used in China. Our results, together with those from other populations, may be of interest to hospitals and lens manufacturers.

Prevalence of WTR corneal astigmatism decreased with increasing age, while prevalence of ATR corneal astigmatism rose. Other studies have reported a similar age-related change in astigmatism axis [10, 13, 42]. This change has been attributed to extraocular muscle tension, visual feedback, corneal tissue degeneration, reduction in eyelid pressure and effects of intraocular pressure on corneal curvature [43]. These findings may help ophthalmologists take into account the effects of aging on the age-related change of astigmatism axis when selecting toric lenses.

AL in our population correlated positively with ACD, as reported in other work [10, 15, 44], while it correlated negatively with K, as reported previously [10, 14, 15, 45]. The positive correlation with ACD may reflect a tendency of shorter eyes to have smaller anterior chambers. The negative correlation with K may reflect a tendency of shorter eyes to have steeper corneas and longer eyes to have flatter corneas [45], suggesting an emmetropic mechanism.

While the present study appears to be the first description of ocular biometric characteristics in such a large population of cataract surgery candidates from western China, we cannot exclude selection bias because ours is a hospital-based population from a single medical center. This is particularly important to keep in mind when speculating about the reasons for the high prevalence of severe axial myopia in our patients, particularly younger ones. The fact that we examined 6933 patients suggests that our findings may be relevant to the broader population of individuals with cataracts in western China, although the IOL Master system can fail in 36–38% of patients with dense or posterior subcapsular cataracts [46, 47], and we excluded patients with severe cataracts. In addition, the retrospective nature of our study means that some relevant biometric parameters were not measured, limiting the analyses that we could perform. For example, cataract grading scale was unavailable for many patients in this retrospective study, and this parameter can influence ocular biometric characteristics [6, 48].

## Conclusion

This study provides data on ocular biometric parameters and their relationships in a large, representative population of cataract patients at least 50 years old from western China. The present work helps expand the reference database on ocular biometric characteristics of Asian cataract populations. It also highlights the need for further studies into the factors that may contribute to the high prevalence of severe axial myopia.

## Abbreviations

ACD: Anterior chamber depth
AL: Axial length
ATR: Against-the-rule
CA: Corneal astigmatism
K: Keratometric power
K1: Minimal keratometric power
K2: Maximal keratometric power
WTR: With-the-rule
